# Design of an Interactive Exercise and Leisure System for the Elderly Integrating Artificial Intelligence and Motion-Sensing Technology

**DOI:** 10.3390/s25072315

**Published:** 2025-04-05

**Authors:** Chao-Ming Wang, Cheng-Hao Shao, Yu-Ching Lin

**Affiliations:** 1 Department of Digital Media Design, National Yunlin University of Science and Technology, Douliu 64002, Taiwan; b85223g@gmail.com; 2 Graduate School of Design, National Yunlin University of Science and Technology, Douliu 64002, Taiwan; shaoch108@gmail.com; 3 Department of Information Management, Chaoyang University of Technology, Taichung 41349, Taiwan

**Keywords:** elderly individuals, artificial intelligence, interactive motion sensing, exercise and leisure system

## Abstract

In response to the global trend of population aging, the issue of providing elderly individuals suitable leisure and entertainment has become increasingly important. In this study, it aims to utilize artificial intelligence (AI) technology to offer the elderly with a healthy and enjoyable exercise and leisure experience. A human–machine interactive system is designed using computer vision, a subfield of AI, to promote positive physical adaptation for the elderly. The relevant literature on the needs of the elderly, technology, exercise, leisure, and AI techniques is reviewed. Case studies of interactive devices for exercise and leisure for the elderly, both domestically and internationally, are summarized to establish the prototype concept for system design. The proposed interactive exercise and leisure system is developed by integrating motion-sensing interfaces and real-time object detection using the YOLO algorithm. The system’s effectiveness is evaluated through questionnaire surveys and participant interviews, with the collected survey data analyzed statistically using IBM SPSS 26 and AMOS 23. Findings indicate that (1) AI technology provides new and enjoyable interactive experiences for the elderly’s exercise and leisure; (2) positive impacts are made on the elderly’s health and well-being; and (3) the system’s acceptance and attractiveness increase when elements related to personal experiences are incorporated into the system.

## 1. Introduction

### 1.1. Research Background and Motivation

#### 1.1.1. Research Background

The global aging population has been steadily increasing in recent years. In Taiwan, for example, by the end of March 2008, 14.05% of the population was aged 65 and older, officially marking the onset of an aging society. With low birth rates, many older adults now face the challenge of living alone, making home safety and suitable leisure activities critical issues. Upon retirement, seniors often experience a shift in focus, requiring emotional adjustment and the need to find new interests and life goals.

Although previous studies have suggested various leisure activities for the elderly, traditional ones, such as chess or walking in parks, remain dominant compared to those enjoyed by younger generations. In 1997, the World Health Organization defined quality of life as an individual’s perception of their position in life, encompassing personal goals, expectations, standards, and concerns. To better meet the needs of the elderly, it is essential to explore innovative leisure activities that incorporate modern technology to enhance their physical health, as well as their overall quality of life [1].

#### 1.1.2. Research Motivation

As the global aging population grows, elderly individuals face challenges such as home safety, adjusting to a new life focus, and engaging in meaningful leisure activities. Since traditional leisure options dominate, it has become urgent to explore how modern technology can better address their evolving needs [2]. With retirement prompting significant lifestyle changes, the rapid development of technology presents new opportunities to enhance their quality of life.

This research aims to explore how artificial intelligence (AI) can create innovative, enjoyable, and health-promoting leisure activities to improve the quality of life for the elderly [3]. Specifically, the study seeks to develop a human–machine interactive system that enables elderly individuals to engage in exercise and leisure using AI techniques like object detection and interactive motion sensing. The system’s impact on the elderly will be evaluated statistically through prototype development and experience activities in elderly-related settings, with a focus on its effects on the physical health, where “physical health” in this study is defined as the “normal functioning of the body”. Accordingly, “improving or promoting the physical health” mentioned in the sequel means “having better uses of the functions of the user’s body”.

Additionally, the feasibility of integrating technology into leisure activities for the elderly will be thoroughly explored, providing valuable insights for future research in this field.

### 1.2. Literature Review

#### 1.2.1. Needs of Aging for the Elderly

As individuals age, their physical and mental functions gradually decline, affecting motor, perceptual, and cognitive abilities, which in turn impact daily life. Huang [4] identified four key indicators of aging: loss of reproductive ability, graying hair, reduced physiological functions, and the onset of chronic diseases. Aging is a complex process influenced by genetics, lifestyle, and health, leading to a decline in the ability to perform daily activities. However, appropriate physical activity can help maintain organ function and delay aging [5]. Aging is not solely driven by disease—factors like reduced physical agility, external response abilities, weight loss, and changes in social and health perceptions also play a role [6]. Elderly individuals often face difficulties using technology due to these age-related changes. Chen [7] noted that the gap in using technological products is wider for the elderly, often due to physiological factors. However, if designers consider aging-related needs, they can create products better suited to the elderly’s physiological and psychological conditions [8].

As the aging population grows, gerontology research increasingly addresses not only the physiological aspects of aging but also the psychological challenges faced by the elderly, aiming to help them live healthier and more fulfilling lives [9]. Rowe and Kahn [10] defined successful aging as involving (1) reducing disease and disability, (2) maintaining cognitive and physical functions, and (3) active participation in daily activities. Baltes and Baltes [11] viewed aging success as a psychological adaptation process, encompassing (1) selection, (2) optimization, and (3) compensation, known as the SOC model. Lin [12] expanded these ideas, defining successful aging as the ability to adapt well to aging, emphasizing physical health, psychological well-being, and maintaining strong family and social relationships to enjoy life in old age.

Successful aging is shaped by individual choices and behaviors, driven by personal autonomy. Franklin and Tate [13] emphasized that life satisfaction is key to successful aging. Regular exercise helps reduce anxiety, depression, and negative emotions, boosts self-esteem, and enhances cognitive function, thereby improving life satisfaction [14]. The concept of “active aging”, introduced in Singapore, encourages ongoing learning and embodies the spirit of aging without feeling aged. Education can delay physical aging and prevent psychological and social decline, promoting successful aging [15].

In addition to active learning, leisure activities play a vital role in successful aging. Li and Gao [16] found a strong positive correlation between leisure participation and successful aging. Heintzman and Mannell [17] showed that leisure activities benefit physical and mental health, skill development, stress relief, and social connections. Yan and Guo [18] observed that leisure education helps elderly individuals improve health, meet personal needs, refocus life, reduce stress, and gain enjoyment, promoting psychological, physiological, and social benefits. Hsieh [19] emphasized that engaging in meaningful activities and maintaining strong family and social ties are essential for successful aging, and various positive approaches to aging contribute to this process [20].

In response to the aging population trend, countries worldwide have implemented strategies to help elderly individuals age with dignity, health, and happiness. The concept of active aging encourages regular participation in physical leisure activities, promoting successful aging and addressing the challenges of growing older.

#### 1.2.2. Exercise and Leisure Activities for the Elderly

After retirement, elderly individuals who stay at home for long periods need activities that refresh both their body and mind. The International Society for Gerontechnology defines gerontechnology as the design of technologies and environments that enable elderly individuals to live independently, healthily, comfortably, and safely while participating in society. Engaging in activities promotes better health for the elderly, as staying home without engaging in daily activities can hinder their sense of well-being. In contrast, social activities help foster a sense of health and well-being [21]. Lin [22] categorized elderly leisure activities into three types: solitary, social, and fitness-oriented. Hughes [23] noted the shift from tangible recreational products like books and tapes to cloud-based media like e-books, music, and videos.

Lian [24] highlighted that leisure activities improve life satisfaction, emotional well-being, physical fitness, and slow the decline in physical functions for elderly individuals. Digital interactive behaviors, in turn, rely on systems that offer real-world models for learning digital operations [25]. Unlike younger individuals, the elderly are slower in both physical activity and visual processing, requiring entertainment options tailored to their capabilities. Caprani et al. [26] found that elderly individuals perform better with touchscreens than other input devices and prefer them. To improve adoption rates, Pal et al. [27] suggested designing systems with ease of operation in mind, using user-friendly interfaces and simple button operations. Thus, touchscreens should be prioritized in designing such devices.

#### 1.2.3. Orange Technology and Applications

Wang [28,29] introduced the concept of orange technology, which emphasizes human-centeredness and humanitarian care, focusing on developing technologies that enhance health, happiness, and well-being. The color orange, a mix of red and yellow, symbolizes brightness, health, happiness, and warmth. Liu and Kao [30] described orange as a balance between the energy of red and the liveliness of yellow, associated with joy and sunlight. In color theory, orange represents passion, charm, creativity, determination, and achievement, evoking warmth and inspiration.

As detailed by Wang [31], Liu and Chen [32], and Chen [33], orange technology encompasses three main areas, health technology, happiness technology, and care technology, outlined as follows. (A) Health technology includes (a) enhancing the health or quality of life for the elderly, (b) integrating computer communications and medical systems, and (c) building cloud platforms for health promotion, disease prevention, telemedicine, mobility, and home care. (B) Happiness technology involves (a) personal and national happiness indices, (b) methods to measure happiness, and (c) applying technology to improve happiness levels. (C) Care technology focuses on designing innovative products and systems that promote human care and connection.

The application of AI in the home domain has expanded significantly, encompassing areas from security monitoring to smart assistants. Today, nearly all household systems are integrated with AI, with four main categories identified [34]: (1) entertainment and information, (2) automated control, (3) security monitoring, and (4) healthcare. Of these, entertainment and information applications are already embedded in homes, and future developments are expected to focus on the latter three categories.

Examples of current smart home applications include facial recognition security locks, smart assistants, and care robots. Existing systems can be classified into five dimensions based on usage: health monitoring, environmental monitoring, companionship, social interaction, and entertainment [27]. Xu and Bai [35] noted that children working away from home often have limited communication with elderly parents, and the greatest risk for the elderly is not just health concerns, but also isolation and loneliness.

The demographic structure of the elderly population is shifting, with an increasing proportion of elderly individuals, leading to an aging society. After 65, most people transition from the workforce to family life, gradually moving from independence to requiring assistance with daily activities. To ease the burden on caregivers, the market is seeing a rise in care robots. Sharkey and Sharkey [36] identified three main functions of care robots: (1) assisting the elderly or caregivers with daily tasks, (2) monitoring the elderly’s behavior and health, and (3) providing companionship. These robots not only address physiological care but also cater to psychological needs. Additionally, smart assistants, integrated with the Internet of Things (IoT), manage household information, enable remote control of appliances, and provide reminders, such as weather forecasts or traffic updates, preventing inconveniences when leaving the house.

Several existing cases of orange technology applications are analyzed and compared in this study from the aspects of human–machine interfacing format, AI technique type, and interaction scheme, as shown in Table 1.

#### 1.2.4. AI and Computer Vision Technology

Artificial intelligence (AI) is a technology that enables computers to simulate human-like thinking and perform specific tasks on behalf of humans. The concept was first introduced by John McCarthy in a 1956 research project on AI, marking the emergence of AI as a distinct academic discipline [40].

Before 1956, AI was in its early stages, with scientists working to develop machines capable of performing human tasks. A key contribution came in 1936 when Alan Turing published his paper “On Computable Numbers, with an Application to the Entscheidungsproblem [41], introducing the “Turing Test” and laying the foundation for modern computers and AI theory. In 1943, Warren Sturgis McCulloch and Walter Harry Pitts Jr. published “A Logical Calculus of Ideas Immanent in Nervous Activity”, introducing artificial neural networks (ANNs) and demonstrating their mathematical relationship to logical expressions, inspiring further research in the field [42].

AI became an established research area only after John McCarthy formally defined it, marking the beginning of its first golden era from 1956 to 1970. During this period, major advancements included machine learning, expert systems, and natural language processing. In 1957, American psychologist Frank Rosenblatt proposed the Perceptron model, and by 1960, it was applied in the Mark 1 Perceptron system to distinguish gender in photographs. This invention was pivotal in the AI field and laid the foundation for modern neural network design [43].

AI entered its second golden era from 1980 to 1990, with breakthroughs such as the Hopfield Neural Network introduced in 1982, advancing speech recognition technologies. After 2006, advancements in graphics card technology and expanded storage capacity significantly improved hardware parallel processing, enabling rapid access to vast data. The introduction of deep learning in 2012 led to major breakthroughs in speech recognition, computer vision, and natural language processing.

It is noteworthy that computer vision, a subfield of AI, has seen significant development over the past two decades and is now widely applied across various domains, including industry, healthcare, and transportation. This field focuses on enabling machines to “see”, often referred to as machine vision. By using cameras and computational systems, computer vision substitutes human visual perception, allowing for the identification, tracking, and measurement of objects, while mimicking human cognitive processes to interpret and recognize images. The core principle of computer vision is to simulate human vision using cameras that capture images and send them to a computer system for real-time processing. The system analyzes these images frame by frame, using algorithms like image segmentation, smoothing, and edge sharpening, which enhance the computer’s ability to process image content efficiently.

One important technique in computer vision is the deformable part model (DPM), which represents an object as a combination of parts. For instance, it models a human as a combination of the head, body, arms, and legs. Felzenszwalb et al. [44] found that the DPM is a more accurate method for object detection compared to traditional hand-crafted feature approaches, such as sliding window feature extraction followed by classification. The model enhances the use of histogram of oriented gradients features and introduces both global and local models, significantly improving object detection accuracy. However, the DPM has drawbacks, including complex features, slower computation speed, and reduced performance when detecting objects that undergo rotation or stretching.

**Figure 1:**
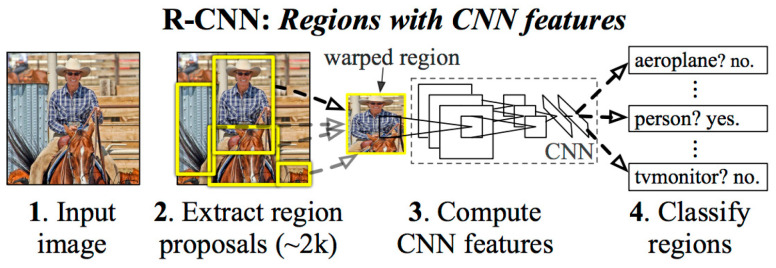
The region-based convolutional neural network (R-CNN) architecture proposed by Girshick et al. [45].

Another computer vision method is the region-based convolutional neural network (R-CNN), proposed by Girshick et al. [45]. This method applies the selective search algorithm to generate candidate regions, which are then processed through a CNN to extract desired features. The extracted features are sent to multiple support vector machine (SVM) classifiers, and regions with high classification accuracy are retained as the final object detection regions. The R-CNN process generates approximately 2000 candidate regions from the original image through selective search. Each candidate region requires CNN feature extraction and SVM classification, resulting in significant computational load and slower detection speeds. However, the R-CNN method offers the advantage of substantially improved detection accuracy and introduces a deep learning-based framework for object detection. An architecture of the R-CNN is shown in Figure 1.

**Figure 2:**
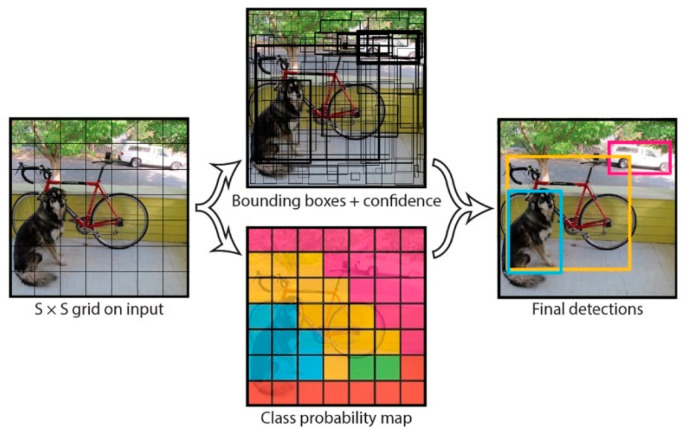
An illustration of the YOLO model proposed by Redmon et al. [47].

A third technique in computer vision is the use of the YOLO (You Only Look Once) algorithm [46] for real-time object detection. The algorithm divides an image into a grid, and detects and processes objects within each grid cell, as shown in Figure 2. Redmon et al. [47] highlighted three advantages of the YOLO algorithm: (1) it enables real-time image processing with less than 25 milliseconds of delay, (2) it performs comprehensive image inference, reducing background error rates to less than half of those in the fast R-CNN algorithm, and (3) it learns generalized representations of objects, making it less prone to errors when processing new or unseen images. However, the YOLO algorithm also has drawbacks, including difficulty in detecting tightly clustered small objects (e.g., flocks of birds) and challenges in generalizing objects with uncommon aspect ratios.

Currently, YOLO has also been applied in elderly care, primarily for fall detection among elderly individuals. Sun et al. [48] categorized human postures into four types, standing, sitting, leaning, and falling, using labeled images to generate extensive training data. Lu and Chu [49] proposed three methods for fall detection: wearable devices, environmental sensors, and image recognition. For wearable devices, tri-axial accelerometers placed on the elderly person’s body collect movement speed changes, determining a fall when acceleration exceeds a preset threshold [50]. Regarding environmental sensors, Su et al. [51] suggested using ceiling-mounted Doppler radar to detect falls by motion analysis.

Finally, image recognition is one of the most widely used computer vision technologies. Agrawal et al. [52] applied background subtraction to identify foreground objects in an image. By using human body contours and template matching, the individual in the image was classified. The distance between the person’s bounding box and the center of the body was then measured to determine whether a fall had occurred.

The differences in the training models of the YOLO, DPM, and R-CNN techniques are organized in this study, as shown in Table 2. Several existing cases of computer vision-based works for interactive experience are analyzed and compared in this study from the aspects of presentation form, technique used, and interaction scheme, as shown in Table 3.

### 1.3. Research Goal and Process

#### 1.3.1. System Design Concepts

In this study, a review of the relevant literature is conducted to explore the physical and mental functional states of the elderly, as well as potential issues of aging. Key concepts such as active aging and successful aging, along with challenges faced by elderly individuals when using technological products, are also discussed. Based on the case analysis, it is observed that AI and computer vision technologies have become increasingly advanced, benefiting not only interactive exhibitions but also leisure and entertainment for the elderly.

This study aims to design a technology-based exercise and leisure system that enables the elderly to enjoy gaming experiences while benefiting from sensory stimulation, hand–eye coordination, and physical exercise. By integrating AI and computer vision technologies, particularly the YOLO algorithm, into leisure activities, the system can enrich the lives of older adults while helping them maintain a healthy mind and body through appropriate physical activity.

More specifically, several prototype concepts derived in this study for designing such a system are outlined as follows.

(1) Selecting appropriate leisure and entertainment themes for the elderly and using situational simulations to foster a sense of connection to reality during device use.
(2) Designing YOLO algorithm-based recognition techniques for the human–machine interface and integrating Kinect-based skeletal recognition schemes, enabling the system to detect body movements and allowing participants to interact naturally.
(3) Designing motion-based interactions that provide an intuitive mode of engagement, reducing operational difficulty and minimizing the burden on the elderly.
(4) Designing appropriate game interactions to enhance the elderly’s cognitive awareness of life and physical activity, thereby improving their physiological functions through entertainment.

#### 1.3.2. Research Goal

It aims to answer the following research questions in this study.

(1) What are the advantages of integrating AI technologies into exercise and leisure activities for the elderly?
(2) How can target detection and computer vision technologies be incorporated into exercise and leisure activities for the elderly?
(3) How can AI technologies be used to enhance the positive effects of exercise and leisure activities for the elderly?

The research objectives of this study are summarized as follows.

(1) The application of AI technologies in human–machine interaction and their forms of expression will be explored.
(2) The correlation between exercise and leisure activities and the lives of the elderly will be examined, and a system prototype will be constructed.
(3) Current AI applications and cases in the field of leisure and entertainment will be investigated, and system design concepts will be summarized.
(4) According to the literature review on the relation between leisure activities and the elderly, an interactive system will be proposed for the elderly using AI technologies.
(5) Through surveys and user interviews, the usability and effect of the developed system will be explored.

#### 1.3.3. Research Process

**Figure 3:**
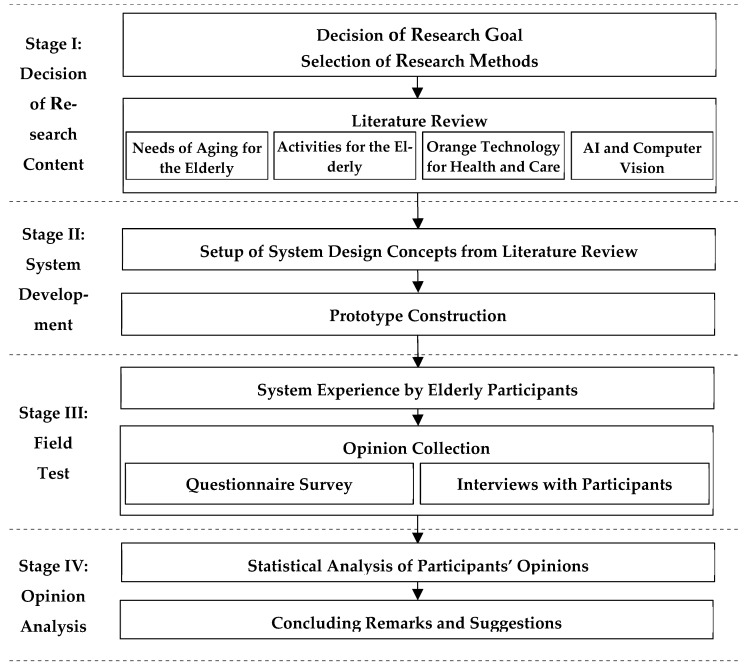
The flowchart of the research process of this study.

The research process of this study is illustrated in Figure 3, which can be divided into four stages as described in the following. More details involved in the process will be described in subsequent sections.

(1) *Stage I: decision of research content*—The research goal and the selection of research methods for this study are determined based on a literature review conducted from four perspectives: (1) the needs of the elderly in aging, (2) leisure activities for the elderly, (3) orange technology for health and care, and (4) AI and computer vision.
(2) *Stage II: system development*—The design concepts of the proposed system are derived from the reviewed literature. Then, a prototype system is constructed accordingly.
(3) *Stage III: Field Test*—The prototype system is tested at a care center, where the elderly are invited to experience the system. Their opinions about using the system are collected by questionnaires and interviews.
(4) *Stage IV: Opinion Analysis*—The users’ opinions are analyzed statistically using the SPSS and AMOS packages, and conclusions are drawn with suggestions for future research also provided.

## 2. Methods

### 2.1. Prototype Development

In this study, a prototype of the proposed interactive exercise and leisure system for the elderly was developed. Following Chi and Guo [56] and based on the methods of Connell and Shafer [57], the development process was divided into seven stages: rapid planning, rapid analysis, rapid development, demonstration and evaluation, prototype revision, requirement approval, and finalization of specification requirements. Additionally, Eliason [58] outlined a four-step prototype development process: requirement analysis, system design, system implementation, and system evaluation.

In this study, the following four steps are followed to build and assess the prototype of the proposed interactive exercise and leisure system.

(1) *Requirement analysis*: Through a literature review, the issues faced by the elderly in leisure activities today are identified. The AI technology and the YOLO algorithm are explored, and the applications of them to leisure activities for the elderly are summarized, culminating in the formulation of the design principles for the system.
(2) *Prototype design*: Based on the derived design concepts, the desired interactive exercise and leisure system for the elderly is designed, with an interactive flowchart being created to illustrate the system’s interaction process.
(3) *Prototype development*: The integration of the motion-sensing technique and the YOLO algorithm are conducted for use in the proposed system for the elderly.
(4) *Prototype evaluation:* A user experience activity is held to evaluate the system’s effectiveness. Public demonstrations, questionnaire surveys, and user interviews are carried out to analyze the users’ feedback and assess the system’s performance.

### 2.2. Questionnaire Survey

The questionnaire survey method is a research approach that collects participants’ opinions through questionnaires, enabling the direct gathering of large amounts of information. Ye and Ye [59] recommended that when designing a questionnaire, specific steps, formats, principles, approaches, and methods should be followed to ensure objectivity, clarity, adequacy, logical consistency, rationality, adaptability, and specificity. Based on statistical analysis results, the effectiveness of the system can be assessed. The implementation steps conducted in this study for the questionnaire survey are as follows.

(1) *Survey time*: After a participant experiences the interactive exercise and leisure system, they are invited to complete a questionnaire, which takes approximately 5 min.
(2) *Survey participants*: Questionnaires will be distributed anonymously to all elderly participants. Each participant’s experience process will last about 10 min, followed by 5 min for completing the questionnaire.
(3) *Survey implementation steps*: (i) Explain the questionnaire items to the participant. (ii) Distribute a questionnaire to each participant. (iii) Request the participant to fill out the questionnaire.

The questionnaire for this study is designed using the technology acceptance model (TAM) [60] and the strategic experiential modules (SEMs) [61], with modifications based on the specific characteristics of this research. The two scales of *system usability* and *user experience* are developed.

After participants interact with the proposed interactive system, quantitative questionnaires are distributed and collected, with valid samples analyzed. Additionally, random sampling of elderly participants are conducted for semi-structured interviews, and verbatim transcripts are analyzed for qualitative validation. The elderly participants’ behavior and experiences with AI in leisure activities will be assessed using the TAM, allowing the impact of AI technology on the elderly to be evaluated. The SEMs will also be employed to assess users’ experiences.

The two scales, system usability and user experience, used in the questionnaire survey are explained in more detail in the following.

(1) *System usability evaluation*: The purpose of this evaluation is to assess the elderly’s acceptance of technology integration in leisure activities, as well as the usability of the system proposed in this study across various aspects. The questionnaire design is primarily based on the TAM [60], with modifications made to suit the specific characteristics of this study. The questionnaire consists of 12 questions, labeled T1 to T12, as shown in Table 4.

(2) *User experience evaluation*: The purpose of this evaluation is to assess the users’ experience and feelings after interacting with the system proposed in this study. The questionnaire design is primarily based on the SEMs [61], with modifications made to suit the specific characteristics of this study. The questionnaire consists of 20 questions, labeled S1 to S20, as shown in Table 5.

The Likert five-point scale [62] was adopted for the questionnaire design, with options ranging from “strongly disagree”, “disagree”, “neutral”, “agree”, to “strongly agree”, assigned the scores of 1, 2, 3, 4, and 5, respectively.

### 2.3. Interviews with Participants

The interview survey method involves direct communication between the researcher and interviewee, allowing for verbal questions and responses that capture objective facts, emotions, attitudes, and value judgments. This approach provides flexibility for interviewees to elaborate on their opinions. In this study, a semi-structured interview approach is used, as described by Flick [63], where topics are introduced with open-ended questions, allowing interviewees to respond based on their knowledge. Lin et al. [64] noted that semi-structured interviews offer a flexible format that enables participants to express their thoughts authentically. This method is employed to explore elderly participants’ experiences with the proposed system.

## 3. Results

### 3.1. System Design

#### 3.1.1. The Design Concept

As mentioned earlier, the proportion of elderly individuals has been steadily increasing in recent years, bringing greater attention to issues related to their daily lives and leisure activities. In response, more communities are organizing group activities for the elderly, encouraging them to leave their homes and engage with others. These initiatives help foster social skills while promoting physical activity and cognitive engagement.

Many current studies focus on applying AI to elderly care and healthcare, with less attention given to exercise and leisure. Therefore, this study aims to incorporate AI technology to develop an interactive exercise and leisure system tailored for elderly individuals.

Called the “Grandpa and Grandma Move”, the system allows elderly users to engage their hand muscles in response to the system’s content and activities. The goal is to stimulate their visual and auditory perception, as well as hand strength, enabling them to follow on-screen instructions while participating in the leisure experience and training their brain. Through this system, it is hoped that elderly individuals will improve their understanding and experience of technology. The system aims to provide a sense of achievement and enjoyment, enhancing the physical function while fostering a positive, active attitude toward aging.

In more detail, AI techniques, including interactive motion sensing with Kinect hardware and real-time object recognition using the YOLO algorithm, are integrated into the proposed system for the elderly. The Kinect is equipped with a dual-lens camera, allowing it to capture images and perform computer vision functions to identify the skeletons of the user for identifying the user’s body actions. Also, the deep learning process carried out in the YOLO algorithm utilizes an image database of various perspectives of balls and tennis rackets constructed in this study to detect the existences of the ball and tennis racket.

Two activities are featured to enhance human–machine interaction: an elastic ball exercise and a tennis exercise. These activities are designed to provide leisure and entertainment for older adults.

The proposed system, which primarily includes a computer host, a TV screen, a webcam, and a Kinect motion-sensing camera, is designed as follows.

**Figure 4:**
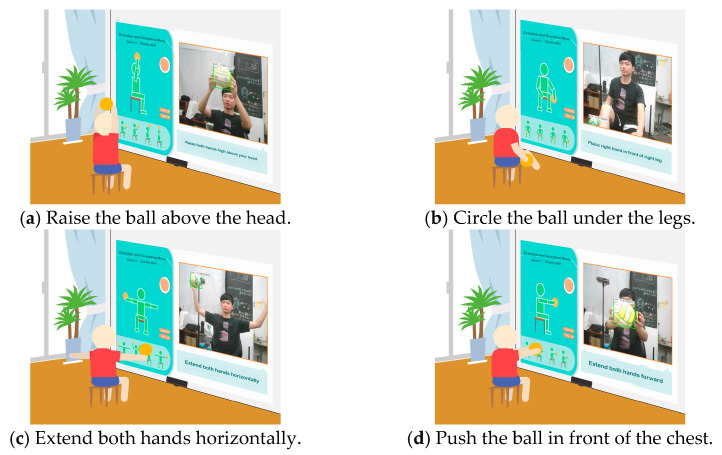
Illustrations of elastic ball exercise game played on the proposed system.

**Figure 5:**
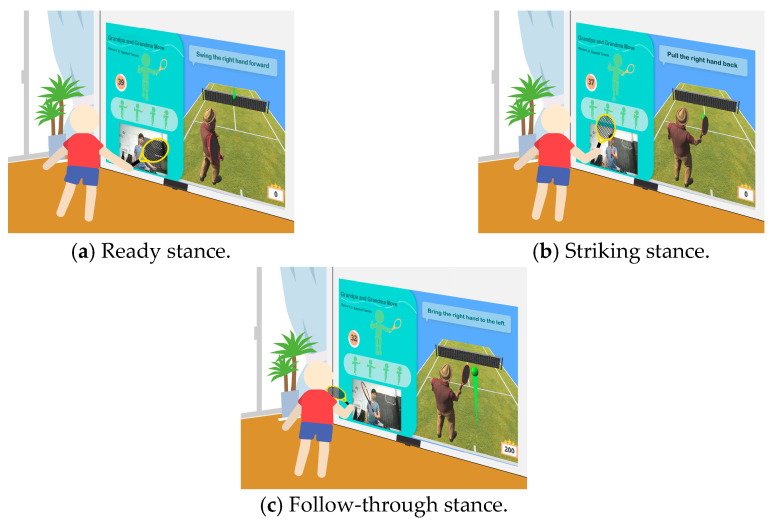
Illustrations of tennis exercise game played on the proposed system.

(1) *Elastic Ball Exercise Experience*: This exercise, based on the elderly person’s past experiences and entertainment activities, is designed to engage their visual and auditory perception as well as hand strength. The participant holds an elastic ball and follows the system’s instructions to perform four actions. The YOLO algorithm detects whether the ball is in the participant’s hand, and if it moves out of range, a prompt appears to ask them to pick it up again for an optimal experience. The process is illustrated in Figure 4.

(2) *Tennis Exercise Experience*: Based on the elderly person’s background, this exercise uses hand and leg muscles to operate the device, aiming to activate visual and auditory perception, as well as leg strength. The participant holds a tennis racket and follows system instructions to swing and hit the ball. The YOLO algorithm detects in real time whether the racket is in the participant’s hand and tracks its orientation. The racket must face the camera; if it is sideways or out of range, a prompt appears on the TV screen to ask the user to reposition the racket for the best experience. The tennis exercise process is shown in Figure 5.

It is emphasized that there is a distinction between the two types of exercise: the elastic ball exercise is performed while sitting, whereas the tennis exercise is performed while standing. This approach offers a more comprehensive physical training experience for the elderly participant.

#### 3.1.2. The Interactive Process

AI technology is integrated into the proposed system, creating an interactive exercise and leisure platform tailored for the elderly. The system follows four main steps: it begins with a standby screen where the user initiates the game by adopting a T-pose. The user then selects either the elastic ball exercise or the tennis exercise to engage in the corresponding activities. The system is designed using Kinect-based motion-sensing interaction to identify the skeletons and the YOLO deep learning algorithm to detect the existences of the ball and tennis racket. A storyboard illustrating the interactive process scenarios is presented in Table 6.

#### 3.1.3. System Architecture

The “Grandpa and Grandma Move” interactive system is developed using Unity, integrating the OpenCV computer vision library and Kinect infrared motion-sensing techniques to track body movements and depth for motion control. The YOLO deep learning algorithm trains two prop models, which are incorporated into the system to identify the user’s selected experience. A Kinect sensor captures the user’s skeletal data, and the system compares these movements to pre-set models via a backend database to verify task completion. The interface is then displayed on a TV screen.

**Figure 6:**
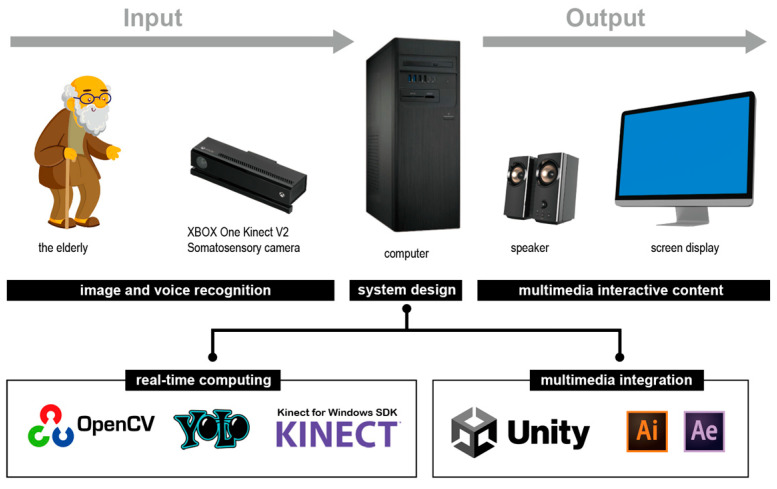
Illustration of the architecture of the proposed system.

In terms of visual design, Illustrator is used to create the main visuals and related elements, which are then imported into After Effects for animation production. The completed animations are placed in Unity for further adjustments and content creation. For 3D models, 3D MAX is used to create the models and adjust character animations, after which the finalized models are imported into Unity for further animation tweaks and coding. This process creates an engaging and entertaining leisure experience for users. The system architecture of the “Grandpa and Grandma Move” is shown in Figure 6.

#### 3.1.4. Main Technologies

The “Grandpa and Grandma Move” interactive system consists of two main components: the motion-sensing interactive interface and the animation content. The motion-sensing interface relies on Kinect’s skeletal detection function, using the user’s skeletal poses to interact with the system. The animation content is generated by Unity, which processes signals from Kinect motion detection and the YOLO algorithm in OpenCV to recognize user actions and trigger corresponding animations. The development environment and hardware/software equipment are outlined in Table 7, with the implemented techniques described in the following sections.

(A) Motion-based Interaction Detection

**Figure 7:**
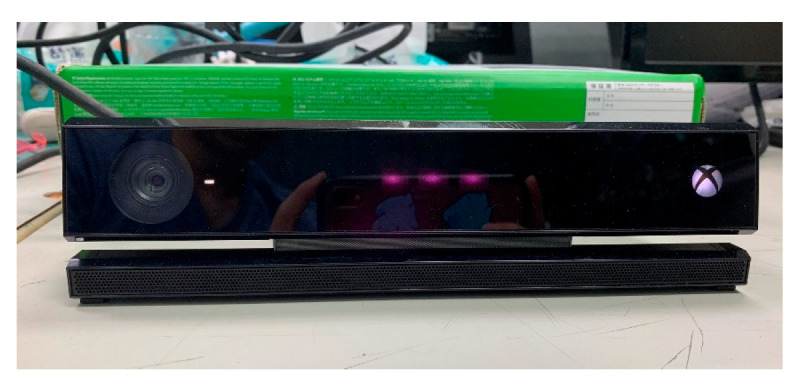
The second-generation Kinect motion-sensing camera used in the proposed interactive exercise and leisure system for the elderly.

The “Grandpa and Grandma Move” interactive system utilizes the Kinect SDK along with the second-generation Kinect motion-sensing camera (shown in Figure 7) to implement motion-sensing operations through computer vision techniques. The Kinect camera includes a depth sensor, an RGB camera, and a microphone array with four units. The depth sensor, which combines an infrared emitter and an infrared camera, uses the time-of-flight (TOF) technique to measure distance based on the time difference between emitted and reflected infrared light. This allows the system to calculate the distance from the object to the depth camera.

The Kinect SDK also supports human tracking and skeletal recognition, enabling the motion-sensing interaction algorithms of the “Grandpa and Grandma Move” system to focus on two main tasks: body skeleton recognition and tracking, as well as limb movement recognition. Details of these functions are described in the following sections.

(1) Body skeleton recognition and tracking

**Figure 8:**
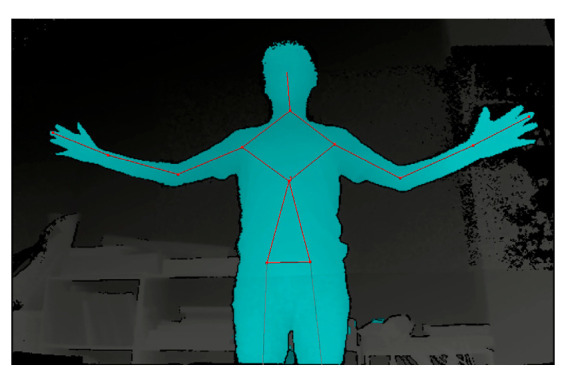
The joint points of a human body tracked by the FAAST.

The FAAST (Flexible Action and Articulated Skeleton Toolkit) and Kinect are used to track the user’s body skeleton and obtain joint coordinates, focusing on the upper body and thigh movements. An illustration of the tracked joint points is shown in Figure 8.

(2) Limb movement recognition

Using the joint positions captured by FAAST, Kinect’s limb recognition function is used to identify the user’s movements and calculate hand bending angles. This algorithm module, shown in Table 8, includes four sets of limb movements, each triggering a corresponding event when specific conditions are met. Additionally, four continuous movements and one tennis swing movement have been designed for elderly users to experience on the “Grandpa and Grandma Move” system, with body recognition descriptions for these five movements provided in Table 9.

The proposed system uses limb movement recognition to control content flow and interactions in both the elastic ball and tennis experiences. By tracking the relative positions of body joint points and the bending angles of hands and legs, the system enables motion sensing to operate the interactive exercise and leisure system. The process is divided into five parts, including two scenarios—the elastic ball and tennis experiences—each implemented through an algorithm as described in Table 10.

(B) Computer Vision and Object Detection

The proposed interactive system integrates AI and multimedia techniques using OpenCV algorithms for object detection. OpenCV’s deep neural network (DNN) module supports all major deep learning frameworks for model training, and from version 3.4.3 onward, it supports the YOLO algorithm. YOLO’s advantage lies in its ability to process images and output predictions through convolutional neural networks (CNNs), offering faster training and computation than other algorithms. In this study, YOLOv3 is used for real-time object detection, with the model trained on the Google Colab platform, which provides free access to high-performance GPUs. The object detection process involves setting up training images, model training, and integrating the detection process, each step of which is described in detail in the following sections.

(1) Setting up training images

**Figure 9:**
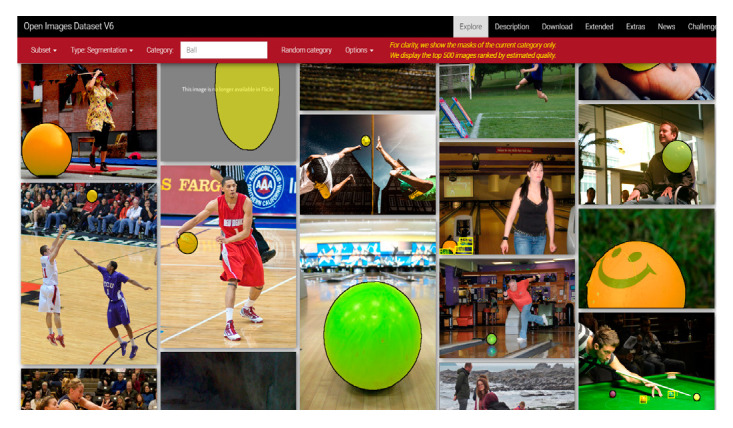
Sample training images obtained from the Open Image Dataset for the YOLO algorithm.

In this study, 1000 pre-labeled training images were collected from the Open Image Dataset using the OIDv4_ToolKit tool. These images were then converted into the format required for the YOLO algorithm. Some sample images are shown in Figure 9.

(2) Model training

To enable real-time detection, the YOLOv3 tiny version was used to reduce recognition accuracy in order to improve the frame rate. Google Colab was used for model configuration and training. The labeled images were uploaded to Google Drive, and file paths were extracted. Pre-trained YOLO model weights were downloaded for reference, and after 8000 iterations, the loss curve stabilized, indicating successful convergence.

(3) Integrating the object detection process

**Figure 10:**
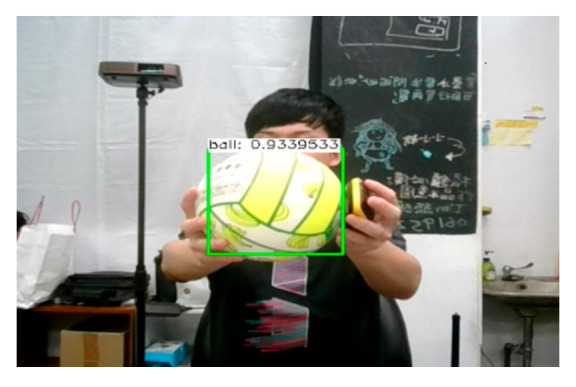
An example of object detection results.

The OpenCV package and trained model files were imported into Unity, where the parameters and weight files were configured for object detection. Using real-time video capture from the camera, the system identifies the elastic ball and highlights it. The model’s confidence in the predicted bounding box is around 93%, indicating a 93% likelihood that the object within the box is a ball. An example is shown in Figure 10.

Object detection, implemented through computer vision in this study, enables interactions in both the elastic ball and tennis experiences. By comparing real-time video captured by the camera with the trained YOLO algorithm model, the system detects the presence of the target object. The object detection process consists of five steps, corresponding to one of three tasks, (1) identifying the object (elastic ball or tennis racket), (2) checking if the elastic ball is in the current frame, and (3) checking if the tennis racket is in the current frame, as outlined by the algorithms in Table 11.

### 3.2. Experimental Design

**Figure 11:**
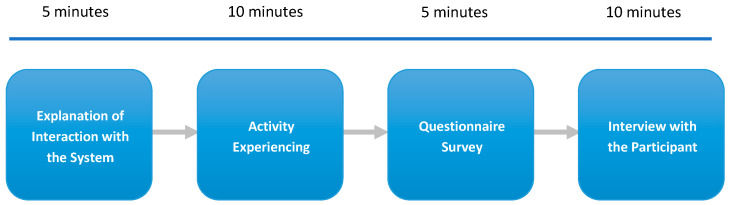
The experimental process for an elderly participant to use the proposed system.

A prototype of the proposed interactive exercise and leisure system for the elderly was developed in this study. After a 5 min briefing on system usage, each participant engaged with the system for 10 min. They then completed a 5 min questionnaire and participated in a 10 min interview. This process allowed for validating the system’s usability and assessing the users’ experiences. The experimental procedure is outlined in Figure 11. The study was conducted across nine sites in Taiwan, specifically in Changhua County and Chiayi City.

### 3.3. Analysis of Questionnaire Survey Results

After using the interactive system, participants filled out a questionnaire. A total of 128 questionnaires were collected, with four invalid due to incomplete responses, leaving 124 valid. The survey gathered participants’ basic information and assessed two key indicators: system usability and user experience.

#### 3.3.1. Sample Structure Analysis

The first part of the questionnaire collected basic participant information, including gender, age, and prior experience with similar technologies (Table 12). The data showed that 20.2% of participants were male, and 79.8% were female. Most participants were aged 55–74 years and 75–84 years, each accounting for 37.9% of the sample. Additionally, 78.2% had no prior experience with interactive systems, indicating limited exposure to similar technologies among the elderly.

#### 3.3.2. Analysis of Reliability and Validity of Questionnaire Survey Results

To evaluate the “system usability” and “user experience” of the interactive system, IBM SPSS 26 and AMOS 23 software were used to perform reliability and validity analyses on the collected questionnaire data. Scores for the two scales were assigned on a 1–5 Likert scale, with each question corresponding to an item listed in Table 4 and Table 5. The results, shown in Table 13 and Table 14, include the minimum (Min.), maximum (Max.), means, and standard deviations (S.D.) for the data. The process of verifying the reliability and validity of the questionnaire data followed five steps, as described below.

(A) Step 1: Verification of the adequacy of the questionnaire dataset

To verify the adequacy of the collected questionnaire data, the Kaiser–Meyer–Olkin (KMO) test and Bartlett’s test of sphericity [65] were used. A KMO value above 0.50 and a Bartlett’s test significance below 0.05 indicate that the data are suitable for further structural analysis.

The KMO and Bartlett’s test results were calculated using data from Table 13 and Table 14 in SPSS, as shown in Table 15. Both KMO values exceeded 0.50, and Bartlett’s significance was below 0.05, confirming that the data are adequate for further analysis.

(B) Step 2: Finding the latent dimensions of the questions from the collected data

The structural analysis for the questionnaire survey aims to categorize the questions of each scale into meaningful subsets, each representing a latent dimension. Exploratory factor analysis (EFA) using principal component analysis and the varimax method with Kaiser normalization was conducted using the SPSS. The results, based on the inputs from Table 13 and Table 14, are presented in Table 16 and Table 17 for the two scales: system usability and user experience.

For the first scale of system usability, the variables T1 through T12, representing the twelve questions asked about this scale, are divided into two groups, FT1 = (T10, T11, T12, T7, T9, T8) and FT2 = (T3, T5, T4, T1, T2, T6), aligning with two latent dimensions termed in this study as “perceived ease of use” and “perceived usefulness”, respectively.

Likewise, for the second scale of user experience, the results are divided into three groups, FS1 = (S6, S19, S7, S2, S20, S4, S5, S3), FS2 = (S14, S16, S15, S17, S18, S13, S12), and FS3 = (S10, S9, S8, S1, S11), corresponding to three latent dimensions termed “sensation and emotion”, “action and connection”, and “cognition and technology awareness”, respectively. These results are comprehensively summarized in Table 18.

(C) Step 3: Verifying the reliability of the collected questionnaire data

Reliability refers to the consistency of a dataset across repetitions [66]. In this study, reliability was assessed using the Cronbach’s α coefficient [67]. A Cronbach’s α coefficient exceeding 0.35 indicates that the data are reliable, and a value surpassing 0.70 signifies high reliability [68]. Using the data from Table 13 and Table 14, the Cronbach’s α coefficients for the two scales and five latent dimensions are provided in Table 19. All coefficients exceed 0.70, confirming that the collected datasets are reliable for further analysis.

(D) Step 4: Verification of applicability of the structural model established with the dimensions

**Figure 12:**
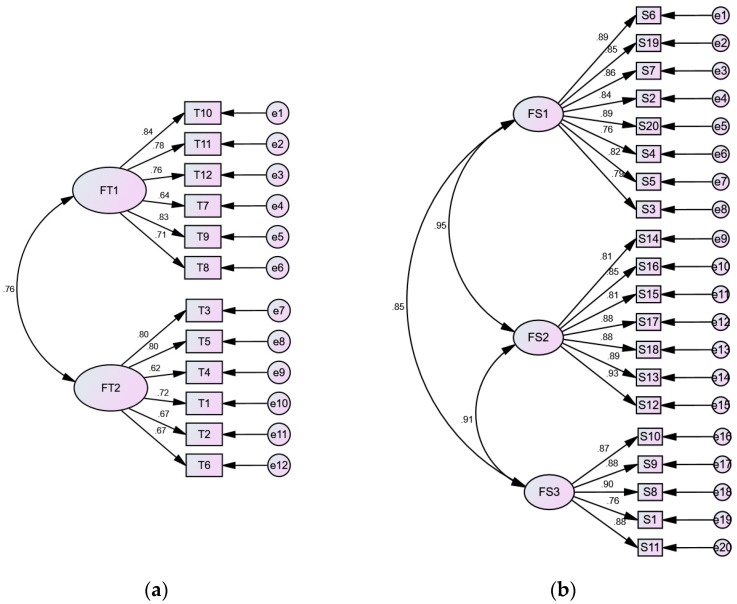
Confirmatory factor analysis (CFA) results using AMOS: (**a**,**b**) are the structural models of scales “system usability” and “user experience” generated through CFA, respectively.

Before validating the questionnaire data, the appropriateness of the structural model based on the latent dimensions has to be verified. This was achieved using confirmatory factor analysis (CFA) with AMOS software, resulting in three structural model graphs, shown in Figure 12. The CFA also generated fit indices for the “system usability” and “user experience” scales, as presented in Table 20. The fit indices χ^2^/df, CFI, and RMSEA for each scale are within acceptable ranges, “1 to 5”, “greater than 0.9”, and “between 0.05 and 0.08”, respectively, indicating a good fit between the structural model and the collected data, as suggested by Hu and Bentler [69].

(E) Step 5: Verification of the validity of the collected questionnaire data

After confirming that the model structures of the two scales fit the questionnaire data, the validity of the data was analyzed. In Figure 12, all factor loading values (standardized regression weights) for the two scales—along the paths from FT1 and FT2 to questions T1–T12, and FS1, FS2, FS3 to questions S1–S20—exceed the threshold of 0.5, indicating good construct validity. This is further supported by the construct validity values for all latent dimensions, computed via EFA and detailed in Table 21, where each value exceeds the 0.6 threshold. These steps collectively verify the reliability and validity of the questionnaire data, allowing for further analysis of each latent dimension.

#### 3.3.3. Analysis of Questionnaire Data About the Scale of System Usability

The usability scale in this study was designed to assess participants’ perceptions of the AI-based system’s usability and acceptance, based on the TAM and its dimensions of “perceived ease of use” and “perceived usefulness”. A brief summary of the results is provided below.

(A) Data analysis for the latent dimension of “perceived ease of use”

The question items T10, T11, T12, T7, T9, and T8 focus on “perceived ease of use”, assessing the system’s simplicity and ease of operation. These items examine participants’ perceptions of factors like operational simplicity, interface clarity, ease of learning, and usage fluency. The analysis of this dimension is presented in Table 22, with key findings summarized below.

(1) The average scores for T10, T11, and T12 range from 4.66 to 4.71 with 100% agreement, indicating the system is easy to operate and interactive.
(2) T9 has an average score of 4.71 and a 99.2% agreement rate, suggesting the interface design meets user needs, though further optimization is recommended.
(3) T7 and T8 received 98.4% agreement, with some users requesting additional learning support, suggesting the inclusion of tutorials or more application scenarios.

The survey results show users have highly positive views of the system’s convenience, interactivity, and interface, with minor improvement opportunities (e.g., T7 and T8) suggesting the need for additional support for new users.

(B) Data analysis for the latent dimension of “perceived usefulness”

The question items T3, T5, T4, T1, T2, and T6 focus on the system’s contributions to physical health, including the coordination of hands, feet, and eyes, as well as its perceived usefulness in promoting health and exercise. These items assess aspects such as coordination improvement, health benefits, relaxation, and willingness to continue using the system. The analysis of the latent dimension “perceived usefulness” is presented in Table 23, with the following key points summarized.

(1) T3, T4, and T5 scored above 4.70, with T5 scoring the highest at 4.80, indicating significant improvement in hand–foot movement and coordination.
(2) T1 and T6 received scores of 4.75 and 4.76, with a 99.2% agreement rate, showing strong recognition of the system’s health benefits and acceptance of continued use.
(3) T2 received a 100% agreement rate, reflecting unanimous satisfaction with the system’s ability to make recreational exercise easier and more enjoyable.
(4) T4 had a slightly lower agreement rate of 97.6%, suggesting that enhancing foot movement and adding health data tracking could improve the user experience.

The results show that users perceive the system as highly effective for enhancing exercise, providing health benefits, and offering a relaxing experience, with a strong willingness to continue using it. Further improvements in foot movement and health features are recommended.

#### 3.3.4. Analysis of Questionnaire Data About the Scale of User Experience

The user experience scale in this study aims to understand participants’ experiences after interacting with the AI-based system. The evaluation scale was designed with reference to Schmitt’s experiential strategy module [61]. It includes three latent dimensions: “sensation and emotion”, “action and connection”, and “cognition and technology awareness”. A brief summary of the analysis of these three latent dimensions is as follows.

(A) Data analysis for the latent dimension of “sensation and emotion”

The question items S6, S19, S7, S2, S20, S4, S5, and S3 focus on participants’ visual, auditory, and emotional responses, such as vitality, affinity, and pleasure. These are classified as “sensation and emotion” experiences and align with the “sense” and “feel” modules in SEMs theory, which emphasize emotional responses to sensory stimuli. The analysis of this latent dimension is presented in Table 24, which reveals the following points.

(1) The average scores for all items of this latent dimension ranged from 4.68 to 4.84, with over 98% of participants agreeing, indicating positive evaluations of visual, auditory, and emotional responses.
(2) S19 received the highest score of 4.84, reflecting the participants’ strong agreement on the system’s entertainment value and interactive appeal, while S20 scored 4.79, indicating the system’s success in motivating the participants to explore technology used in exercise and leisure activities.
(3) S7 and S3 scored 4.73 and 4.72, respectively, confirming the key role of audiovisual feedback in sustaining the engagement and enhancing participation of the elderly users.

The results demonstrate that the system excels in offering sensory stimulation and emotional experiences, boosting satisfaction and engagement, and achieving the goals of bringing the “sensation and emotion” experience to the participants.

(B) Data analysis for the latent dimension of “action and connection”

Question items S14, S16, S15, S17, S18, S13, and S12 focus on participants’ social behaviors (e.g., recommending and sharing) and their intention to continue using the system (e.g., re-engagement and increased motivation for leisure and entertainment). These can be classified as “action and connection” experiences, aligning with the “action experience” and “relational experience” modules in SEMs theory, which emphasize user behavior and societal connections. The analysis of the “action and connection” dimension is shown in Table 25.

(1) S14 and S15, both with an average score of 4.80 and a 100% agreement rate, indicate strong social propagation effects and participants’ strong intentions to recommend and share the system.
(2) S16, with the highest average score of 4.81 and a 100% agreement rate, reflects participants’ very high willingness to reuse the system and their clear intention to continue using it.
(3) S17, with an average score of 4.77, and S18, with an average score of 4.83, were highly rated, demonstrating that the system effectively motivates participants to engage in more beneficial activities, enhancing their motivation for leisure, entertainment, and health.
(4) S12, with an average score of 4.77 and a 98.4% agreement rate, shows that participants recognize the system’s role in enhancing their perception of leisure entertainment and its value.

The questionnaire results reveal strong agreement with the “action and connection” experience, indicating that the system promotes social behaviors, enhances motivation, and increases the perceived value of leisure and entertainment, successfully meeting the goals of the module design.

(C) Data analysis for the latent dimension of “cognition and technology awareness”

The items S10, S9, S8, S1, and S11 focus on participants’ understanding of technology, changes in attitudes, and perceptions of the interactive system after the experience. These can be categorized as “cognition and technology awareness” experiences. Additionally, they align with the “thinking experience” module in SEMs theory, which emphasizes encouraging users to think and inspiring their cognition. The analysis of the latent dimension “cognition and technology awareness” is presented in Table 26. Based on this table, the following points are summarized.

(1) S10, with an average score of 4.70, and S11, with an average score of 4.71, received high ratings and over 96% agreement, indicating that the system effectively enhances participants’ understanding of and attitudes toward technology, improving their awareness.
(2) S9, with an average score of 4.71, shows that participants developed a higher acceptance of and willingness to explore technology, highlighting the system’s potential to inspire engagement with new technologies.
(3) S8 received the highest average score of 4.81, with a 99.2% agreement rate, reflecting strong recognition of the tech–entertainment combination. Additionally, S1 scored 4.72, suggesting the system’s high appeal and entertainment value based on participants’ perceptions.

The questionnaire results show that participants highly agree with all aspects of the “cognition and technology awareness” experience. The system not only successfully enhances technological awareness and changes attitudes but also excels in interactivity and entertainment, achieving the anticipated goals of the system design.

### 3.4. Analysis of Results of Interviews with Participants

After the interactive system experience, 10 elderly participants were invited for interviews, which were conducted using a semi-structured format. The interview content was divided into three areas: (1) system interface operation, (2) experience perception, and (3) views on the integration of AI into leisure activities for the elderly. The first area focused on participants’ opinions about the system’s interface and how smoothly the learning process went. The second area explored the participants’ feelings and experiences with the overall system and its content. The third area discussed participants’ thoughts on the introduction of AI into leisure activities for elderly people.

#### 3.4.1. Record of User Interviews

Table 27 presents the content and organization of the user interview results, listed in order according to the areas discussed. The invited interviewees are labeled P1~P10.

#### 3.4.2. Summary of Interview Results

Based on the participant feedback, AI technology in elderly leisure activities is viewed as a positive innovation with a strong appeal and potential in senior entertainment. The participants showed a positive attitude toward the technology and expressed a desire for more similar activities in the future to improve their quality of life and sense of involvement. The following are more detailed findings.

(1) *System Interface is easy to understand and operate*—Participants found the interface clear and intuitive, with most reporting smooth learning and operation, although some initially faced difficulties.
(2) *The interaction schemes are innovative, promoting physical health*—The novel, body movement-based interaction effectively trained physical functions while fostering emotional connections, increasing happiness and engagement.
(3) *The exhibition and design of the proposed system received high praise*—The exhibition layout and digital content were engaging and suitable for elderly users, with friendly staff and enjoyable game content enhancing the experience.
(4) *AI Technology enhances interaction and willingness to participate*—AI technology made activities more interactive and interesting, sparking interest and openness to new technologies, and encouraging eagerness for future activities.
(5) *Future development suggestions are proposed*—Future development suggestions are proposed: Participants expressed a desire for more frequent activities to benefit their health and entertainment and suggested diversifying content and gameplay to better meet their needs.

In short, the proposed system provides elderly users with an enjoyable, health-promoting experience, receiving highly positive feedback on the interface, interaction design, and their willingness to engage.

## 4. Discussion and Conclusions

### 4.1. Discussion

The interactive exercise and leisure system for the elderly developed in this study is described, along with details of public demonstrations, experimental processes, user interviews, questionnaire surveys, and result analysis. While suggestions for improvements in the elderly-focused design and visual interface were provided by users, overall feedback was mostly positive, with the system being perceived as more innovative and entertaining compared to previous leisure activities after the introduction of AI techniques.

The reliability and validity of responses to the “system usability” and “user experience” questionnaire items were found to exceed standard thresholds, confirming the trustworthiness of the data. Questionnaire results indicated that the average score for most items was above 4 on a 1-to-5 scale, reflecting positive responses from elderly participants regarding their experience with and acceptance of the AI-powered interactive system.

In more detail, the user interviews and questionnaire surveys revealed the following findings:

(1) The interactive system was found to provide positive emotional experiences, such as happiness and joy, for the elderly.
(2) The system’s gameplay was considered novel and easy to engage with, making it interesting and attractive to elderly users.
(3) The elderly expressed a positive attitude toward the integration of AI, indicating good acceptance of AI-based leisure systems.
(4) The system was believed to enhance physical activity and hand–eye coordination.
(5) The elderly were able to easily understand the system’s interaction methods, reflecting a positive experience.
(6) The system’s usability and ease of use averaged above 4.48 on a 1-to-5 scale, showing it is well suited for elderly users.

Furthermore, the reasons why the proposed system works satisfactorily for the users have been reviewed in this study, which are listed in the following.

(1) Several sports were tested during the system design process, and ultimately, ball manipulation games and racket-based tennis were selected for their ease of play and suitability for elderly users.
(2) Elderly users prefer ball games over tennis because the simpler and symmetrical shape of the ball makes it easier to detect by the YOLO algorithm, whereas the racket in tennis adds complexity.
(3) The use of the real-time, versatile AI algorithm YOLO lowers the difficulty threshold, making it easier for elderly users to progress through the games, thereby increasing their willingness to engage with the system.
(4) These AI-driven, easy-to-play games, combined with media prompts, enhance elderly users’ interest in continuous gameplay, effectively achieving the system’s goal of improving their physical health.

### 4.2. Concluding Remarks

With technological advancements, interactive technology in elderly leisure and entertainment has become more common. Unlike activities for younger people, systems for the elderly focus on addressing age-related physical and mental challenges and improving interactions with technology. Intuitive interfaces stimulate creativity and learning in the elderly. In this study, AI technologies, including motion sensing and computer vision, are used to guide elderly users through simple actions, helping them engage in exercise and leisure activities while connecting with technology.

A literature review of related cases was conducted to explore AI applications in elderly leisure experiences. Accordingly, design principles were applied to create an AI-based system with motion-sensing and target-detection capabilities. The resulting system, called the “Grandpa and Grandma Move”, offers an intuitive platform that enhances the enjoyment of exercise and leisure activities for elderly users.

After prototype development and public demonstrations, questionnaire surveys and user interviews were conducted to evaluate the system’s effectiveness. Statistical analysis using SPSS and AMOS revealed that the system effectively enhances the leisure and entertainment experience of elderly participants, as confirmed by three key observations described in the following.

(1) *AI technology enhances interactive leisure experiences for the elderly*—An interactive exercise and leisure system for the elderly was designed in this study, and elderly users were fascinated by its motion-sensing technology. It was found through user interviews that the system was considered novel and enjoyable by the elderly.
(2) *The proposed interactive system promotes elderly health and well-being*—Positive feedback was given by the majority of elderly participants in the survey and interviews. The system’s interface was found to be smooth and enjoyable, and improvements in hand–eye coordination and cognitive abilities were reported, contributing to a healthy, active lifestyle.
(3) *Aligning the system with familiar experiences boosts acceptance*—The system was designed to enhance physical functions of the elderly while providing entertainment. Familiar elements, such as elastic ball and tennis exercises, were incorporated, and higher acceptance and engagement were reported, making the system more attractive than traditional leisure activities and encouraging continued use.

### 4.3. Suggestions for Future Research

In this study, an interactive exercise and leisure system suitable for elderly users has been constructed. Due to time and resource limitations, improvements on the system design still can be made for future research. Some suggestions in this regard are listed below.

(1) *The movements required could be simpler and clearer*—The motion-sensing system might benefit from prioritizing simple postures and ergonomic design, considering elderly users’ physical abilities. Additional guidance, such as animation and audio, could be helpful for operation explanations.
(2) *The difficulty of object recognition may need further consideration*—The YOLO algorithm struggles with irregular objects, like the side angle of a tennis ball. To improve recognition, users could interact with objects like the racket from a frontal position, and the design could consider the algorithm’s limitations to enhance interaction diversity.
(3) *Additional visual and auditory feedback could enhance the system*—To improve user experience, more visual and auditory feedback might be added. Additionally, introducing multiplayer modes (cooperative or competitive) could further enrich the interaction.
(4) *The system could be expanded to other elderly leisure environments*—The system could be extended to other elderly environments (e.g., community centers, parks, rehabilitation centers), with adjustments for specific functions, creating a modular system that adapts to different settings.
(5) *Other applications and studies may also be conducted*—The proposed system can be adapted to meet the needs of individuals in recovery and those with disabilities, with adjustments in game content and interaction style to provide new health benefits; the sample size can be expanded as much as possible to improve the representativeness and statistical power of the research results; the case of multi-day system uses may be studied to support additionally the statement of positive impact on the health; and finally, the system usability, which might change over time with prolonged use, may be investigated.

## Figures and Tables

**Table 1:** Analysis of existing cases of orange technology.

Title	Human–Machine Interfacing	AI Technique	Interaction Scheme
LOVOT [37]	Robot	Deep Learning	Developing a unique personality through the user’s touches and interactions.
Temi-The Personal Robot [38]	Robot	Machine Learning	Performing autonomous navigation and adjusting screen angles based on the user’s need.
SmartAll: The First AI Butler for Everyone [39]	Smart Butler	Machine Learning	Recording user behavior through algorithms and reacts accordingly.

**Table 2:** Differences in the training models of the YOLO, DPM, and R-CNN techniques.

	DPM	R-CNN	YOLO
Detection Scheme	Sliding window	Selective search	Full image detection
Detection Accuracy	High	High	Lower
Detection Speed	Slow	Slow	Fast
Generalization	High	Lower	Highest

**Table 3:** Analysis of existing cases of computer vision-based interactive experience works.

Title	Presentation Form	Technique Used	Interaction Scheme
Interactive Basketball Court [53]	Interactive Space	Target detection, tracking, and real-time computing	The camera captures the player’s movements and projects them in sync with the player’s actions.
Continuous Life and Death at the Crossover of Eternity [54]	Interactive Wall	Target tracking and real-time computing	The flowers on the wall bloom through computer processing and change based on the visitor’s actions.
Worlds Unleashed and then Connecting [55]	Interactive Table	Target detection, tracking, and image recognition	When tableware is placed on the table, it interacts and connects with the projected table surface.

**Table 4:** Questions designed for evaluation of system usability in the questionnaire survey.

Label	Question
T1	Using this interactive system helps improve my physical health.
T2	During the experience, it made my leisure exercise easier.
T3	During the experience, it enhanced my hand movements.
T4	During the experience, it enhanced my leg movements.
T5	During the experience, it improved the coordination between my eyes, hands, and feet.
T6	I am willing to continue using the interactive system.
T7	Learning to use the interactive system was easy for me.
T8	I found that the interactive system made leisure exercise simpler for me.
T9	I think the interface of the interactive system is easy to understand.
T10	Operating the interactive system did not require much mental effort.
T11	I think the operation of the interactive system is very intuitive.
T12	The interaction while operating the interactive system was very smooth.

**Table 5:** Questions designed for evaluation of user experience in the questionnaire survey.

Label	Question
S1	During the experience, the overall interaction was very engaging for me.
S2	During the experience, it made me feel a sense of friendliness.
S3	During the experience, it brought me a great visual experience.
S4	During the experience, the music gave me a pleasant feeling.
S5	During the experience, it enhanced my interest in leisure and entertainment.
S6	During the experience, the interaction energized me.
S7	During the experience, the audiovisual feedback attracted me to continue using it.
S8	During the experience, I felt the entertainment aspect of technology.
S9	After the experience, I became more willing to try technology.
S10	After the experience, I gained a better understanding of the uses of technology.
S11	After the experience, it changed my perception of interactive systems.
S12	After the experience, I understood the importance of leisure and entertainment.
S13	I would recommend this interactive system to my friends.
S14	When my friends ask, I will recommend this interactive system.
S15	I would share this experience with my friends.
S16	If given the chance, I would experience this interactive system again
S17	The interactive system has motivated me to engage in leisure activities.
S18	The interactive system strengthened the connection between leisure activities and physical health.
S19	It showed me how body movements can create leisure experiences with the interactive system.
S20	The design of the interactive system made me more willing to engage with technology.

**Table 6:** Storyboard for interactive scenarios.

Step No.	Stage	Interaction Description	Label	Game Interface
**1**	**Standby**	**1.** When there is no user present, the system will play an idle animation.	**1.**	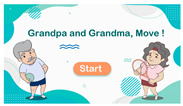
**2**	**Select Game**	**2.** Users can initiate the game by a T-pose and select a desired item (“elastic ball” or “tennis racket”).	**2.**	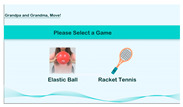
**3**	**Elastic Ball Sports Experience**	**3.1** The user is required to perform four different actions according to the instructions: **(3.1a)** Lift the ball overhead; **(3.1b)** Pass the ball under the legs; **(3.1c)** Switch hands over the head; **(3.1d)** Push the ball in front of the chest. After performing each action five times, the user is required to proceed to the next action.	**(3.1a)**	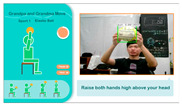
			**(3.1b)**	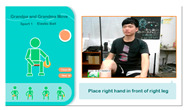
			**(3.1c)**	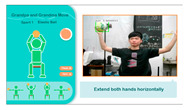
			**(3.1d)**	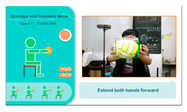
		**3.2** If the user is not holding the ball, the system will display a prompt asking them to pick up the ball. The prompt disappears as the system detects the ball again.	**3.2**	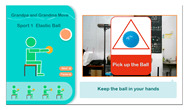
		**3.3** If the user does not perform the correct action within 5 s, the system will still consider the action completed. As all actions are completed, the ending screen will appear.	**3.3**	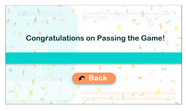
**4**	**Tennis Sports Experience**	**4.1** The user must hit the ball back to the opposite side of the court as many times as possible within a one minute to score points.	**4.1**	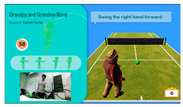
		**4.2** If the racket leaves the screen or the user is not holding it, a prompt will appear, asking the user to pick it up. The prompt disappears once the system detects the racket again.	**4.2**	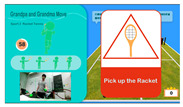
		**4.3** If the user does not perform the correct swing within 10 s, the system will count it as complete, and the ending screen will appear once all actions are performed.	**4.3**	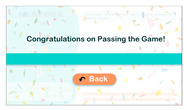

**Table 7:** The environment and hardware/software equipment of the proposed interactive system.

Software (Version Number)		Hardware
Unity 3D (2022.3.20) Adobe Illustrator (2022.23.0) Adobe After Effect (2022.23.0) 3D Max (2022)	FAAST (2012) Google Colab (3.7) OpenCV (4.5.5)	PC TV screen Kinect motion-sensing camera Webcam

**Table 8:** Body movement recognition algorithm module for the proposed “Grandpa and Grandma Move” interactive system.

Label	Function	Algorithm
**G0**	Recognizing limb movements based on the detected target joint points according to specific conditions	**Input:** Depth images and limb movement recognition conditions received by Kinect. **Output:** Completed limb movement event. **Method:** **Step 1:** Use Kinect to detect the user’s limb movements. **Step 2:** If the user’s limb movements meet the pre-set conditions, proceed to Step 3. **Step 3:** Output the limb movement event.

**Table 9:** Limb movement event for the proposed “Grandpa and Grandma Move” interactive system.

Label	Limb Recognition Event	Illustration	Limb Recognition Condition
**G1**	Place both hands in front of the chest	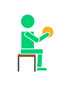	Place both hands approximately 10 cm in front of the chest.
	Raise both hands high	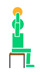	Raise both hands above the head by at least 20 cm.
**G2**	Place the right hand in front of the right leg	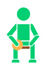	Place the right hand 5 cm in front of the right leg.
	Raise the right leg	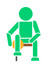	Raise the right leg more than 10 cm and move the right hand underneath the right leg.
	Place the left hand in front of the left leg	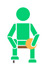	Place the left hand 5 cm in front of the left leg.
	Raise the left leg	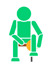	Raise the left leg more than 10 cm and move the left hand underneath the left leg.
**G3**	Extend both hands horizontally	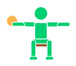	Extend both hands horizontally at shoulder height, forming a “T” shape, with each hand 20 cm away from the shoulder.
	Raise both hands high	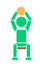	Raise both hands above the head by at least 20 cm.
**G4**	Place both hands in front of the chest	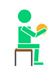	Place both hands approximately 10 cm in front of the chest.
	Extend both hands forward	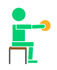	Extend both hands straight out in front, at least 30 cm beyond the chest.
**G5**	Pull the right hand back	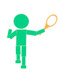	Pull the right hand back, at least 15 cm away from the right shoulder, to prepare for a swing.
	Swing the right hand forward	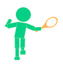	Swing the right hand forward, keeping it at least 20 cm away from the shoulder.
	Bring the right hand to the left shoulder	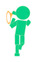	Pull the right hand back to a position 10 cm away from the left shoulder.

**Table 10:** Motion-sensing interactive control algorithm for the proposed “Grandpa and Grandma Move” interactive system.

Label	Function	Algorithm
**C1**	Maintain the T-shape pose and trigger the button event to enter the game selection page.	**Input:** Depth images and T-pose recognition count TC, all received by Kinect. **Output:** Entering the game selection page. **Method**: **Step 1:** Detect if the user is performing the T-pose until it is completed. **Step 2:** If the TC is smaller than 3, increment TC by 1 and proceed to Step 3; otherwise, proceed to Step 5. **Step 3:** If the T-pose is continuously detected every 0.5 s, return to Step 2; otherwise, proceed to Step 4. **Step 4:** Cancel the detected T-pose, and reset TC to zero. **Step 5:** When TC is greater than or equal to 3, trigger the button event to enter the game selection page.
**C2**	Maintain the holding object posture with both hands and trigger the button event to enter the elastic ball experience or tennis racket experience.	**Input:** Depth images and the number HC of times the user performs the two-handed holding object gesture, all received by Kinect. **Output:** Triggering the button event to enter the interactive experience page. **Method**: **Step 1:** Detect if the user is maintaining the two-handed holding object gesture until it is completed. **Step 2**: If HC is smaller than 3, increment HC by 1 and proceed to Step 3; if HC is greater than or equal to 3, proceed to Step 5. **Step 3**: If the two-handed holding object gesture is continuously detected every 0.5 s, return to Step 2; otherwise, proceed to Step 4. **Step 4**: Cancel the two-handed holding object gesture, reset HC to zero. **Step 5**: When HC is greater than or equal to 3, trigger the button event to enter the interactive experience page.
**C3-1**	**Elastic Ball Experience:** “Raise the Ball” Motion Detection.	**Input:** Depth images and the number HUC of times the user raises both hands, all received by Kinect. **Output:** Proceeding with the “Raise the Ball” motion command. **Method**: **Step 1:** Detect if the user is raising both hands to hold the ball until it is completed. **Step 2:** If HUC is less than 5, proceed to Step 3; if HUC is greater than or equal to 5, proceed to Step 5. **Step 3:** If both hands are raised at least 20 cm above the head, increment HUC by 1 and increase the progress bar by 1, then return to Step 2; otherwise, proceed to Step 4. **Step 4:** If both hands are not raised at least 20 cm above the head, HUC does not increase. **Step 5:** When HUC is greater than or equal to 5, proceed to the “Underhand Ball Rotation” experience.
**C3-2**	**Elastic Ball Experience:** “Underhand Ball Rotation” Motion Detection.	**Input:** Depth images, the number RLC of times the right leg is raised, and the number LLC of times the left leg is raised, all received by Kinect. **Output:** Proceed with the “Underhand Ball Rotation” motion command. **Method**: **Step 1:** Detect if the user is performing the “Underhand Ball Rotation” motion until it is completed. **Step 2:** If RLC and LLC are both less than 5, proceed to Step 3; if both RLC and LLC are greater than or equal to 5, proceed to Step 5. **Step 3:** If the right leg is raised at least 10 cm, increment RLC by 1; if the left leg is raised at least 10 cm, increment LLC by 1, and increase the progress bar by 1. Then, return to Step 2; otherwise, proceed to Step 4. **Step 4:** If the right leg is raised less than 10 cm, RLC does not increase. If the left leg is raised less than 10 cm, LLC does not increase. **Step 5:** When both RLC and LLC are greater than or equal to 5, proceed to the “Hand Swap Overhead” experience.
**C3-3**	**Elastic Ball Experience:** “Hand Swap Overhead” Motion Detection.	**Input:** Depth images, the number RHC of times the right hand is raised horizontally, the number LHC of times the left hand is raised horizontally, and the number BHC of times both hands are raised above the head, all received by Kinect. **Output:** Proceed with the “Hand Swap Overhead” motion command. **Method:** **Step 1:** Detect if the user is performing the “Hand Swap Overhead” motion until it is completed. **Step 2:** If RHC, LHC, and BHC are all less than 5, proceed to Step 3; if any of RHC, LHC, or BHC are greater than or equal to 5, proceed to Step 5. **Step 3:** If both hands are raised horizontally at least 20 cm from the shoulders, increment RHC and LHC by 1; if both hands are raised above the head by at least 20 cm, increment BHC by 1, and increase the progress bar by 1. Then, return to Step 2; otherwise, proceed to Step 4. **Step 4:** If the hands are not raised at least 20 cm horizontally from the shoulders, RHC and LHC do not increase. If both hands are not raised at least 20 cm above the head, BHC does not increase. **Step 5:** When RHC, LHC, and BHC are all greater than or equal to 5, proceed to the “Push the Ball in Front” experience.
**C3-4**	**Elastic Ball Experience:** “Push the Ball in Front” Motion Detection.	**Input:** Depth images and the number HFC of times both hands are pushed forward, all received by Kinect. **Output:** Proceed with the “Push the Ball Forward” motion command. **Method**: **Step 1:** Detect if the user is pushing both hands forward until it is completed. **Step 2:** If HFC is less than 5, proceed to Step 3; if HFC is greater than or equal to 5, proceed to Step 5. **Step 3:** If both hands are pushed at least 30 cm forward from the chest, increment HFC by 1 and increase the progress bar by 1. Then, return to Step 2; otherwise, proceed to Step 4. **Step 4:** If both hands are not pushed at least 30 cm from the chest, HFC does not increase. **Step 5:** When HFC is greater than or equal to 5, proceed to the score calculation screen.
**C4**	**Tennis Experience:** “Swing Pose” Motion Detection.	**Input:** Depth images and the position RHP of the right-hand joint received by Kinect. **Output:** Swing motion command. **Method:** **Step 1:** Detect the user’s right-hand position. **Step 2:** If RHP is at least 15 cm behind the right shoulder, enter the swing preparation posture animation, then proceed to Step 3. **Step 3:** If RHP is at least 20 cm to the right of the shoulder, enter the swing posture animation, then proceed to Step 4. **Step 4:** If RHP is at least 5 cm to the left of the left shoulder, enter the follow-through posture animation, then proceed to Step 5. **Step 5:** Complete one full swing motion.
**C5**	Maintain the T-shape pose and trigger the button event to return to the home page.	**Input:** Depth images and the number EPC of times the T-pose body gesture is detected, all received by Kinect. **Output:** Command to return to the home page. **Method:** **Step 1:** Detect if the user is performing the T-pose body gesture until it is completed **Step 2:** If EPC is less than 3, increment EPC by 1 and proceed to Step 3; if EPC is greater than or equal to 3, proceed to Step 5. **Step 3:** If the T-pose body gesture is continuously detected every 0.5 s, return to Step 2; otherwise, proceed to Step 4. **Step 4:** Cancel the T-pose body gesture and reset EPC to zero. **Step 5:** When EPC is greater than or equal to 3, trigger the button event to return to the home page.

**Table 11:** Object detection algorithms for the proposed interactive system.

Label	Function	Algorithm
**R1**	Detect the item held by the user and enter the game corresponding to that item	**Input:** Real-time video captured by the webcam. **Output:** Identify the object held by the user. **Method:** **Step 1:** Capture the user and surrounding environment using the webcam. **Step 2:** Perform grid segmentation on the captured image. **Step 3:** Compare and predict each grid with the trained model. **Step 4:** Select the target that most closely matches the prediction. **Step 5:** Based on the detection results, enter the elastic ball experience or tennis experience.
**R2**	Detect whether the elastic ball stays on the screen	**Input:** Real-time video captured by the webcam, elastic ball. **Output:** Prompt for the disappearance of the target. **Method:** **Step 1:** Capture the user and surrounding environment using the webcam. **Step 2:** Perform grid segmentation on the captured image. **Step 3:** Compare and predict each grid with the trained elastic ball model. **Step 4:** Analyze whether the elastic ball is visible in the frame. **Step 5:** If the elastic ball is not detected for over 5 s, the system will display a prompt asking the user to pick up the elastic ball.
**R3**	Detect whether the tennis racket stays on the screen	**Input:** Real-time video captured by the webcam, tennis racket. **Output:** Prompt for the disappearance of the target. **Method:** **Step 1:** Capture the user and surrounding environment using the webcam. **Step 2:** Perform grid segmentation on the captured image **Step 3:** Compare and predict each grid with the trained tennis racket model. **Step 4:** Analyze whether the elastic ball is visible in the frame. **Step 5:** If the tennis racket is not detected for over 5 s, the system will display a prompt asking the user to pick up the tennis racket.

**Table 12:** Statistical summary of the participants’ basic information.

Basic Data	Category	No. of Samples	Ratio (%)
Sex	Male	25	20.2
	Female	99	79.8
Age	55–64	16	12.9
	65–74	47	37.9
	75–84	47	37.9
	85 and above	14	11.3
Having used interactive motion-sensing systems in the past	Yes	27	21.8
	No	97	78.2

**Table 13:** Statistics of the questionnaire data of scale of “system usability”.

No.	Min	Max	Mean	S.D.	Strongly Agree	Agree	No Opinion	Disagree	Strongly Disagree	Percentage of Agreements
					5 Scores	4 Scores	3 Scores	2 Scores	1 Scores	
					(A)	(B)	(C)	(D)	(E)	(F = A + B)
T1	3	5	4.75	0.453	75.8	23.4	0.8	0	0	99.2
T2	4	5	4.70	0.459	70.2	29.8	0	0	0	100
T3	3	5	4.77	0.457	79.0	19.4	1.6	0	0	98.4
T4	3	5	4.73	0.496	75.8	21.8	2.4	0	0	97.6
T5	3	5	4.80	0.423	80.6	18.5	0.8	0	0	99.1
T6	3	5	4.76	0.449	76.6	22.6	0.8	0	0	99.2
T7	3	5	4.58	0.527	59.7	38.7	1.6	0	0	98.4
T8	3	5	4.70	0.494	71.8	26.6	1.6	0	0	98.4
T9	3	5	4.71	0.473	71.8	27.4	0.8	0	0	99.2
T10	4	5	4.71	0.456	71.0	29.0	0	0	0	100
T11	4	5	4.66	0.475	66.1	33.9	0	0	0	100
T12	4	5	4.69	0.463	69.4	30.6	0	0	0	100

**Table 14:** Statistics of the scale of “user experience” questionnaire data.

No.	Min	Max	Mean	S.D.	Strongly Agree	Agree	No Opinion	Disagree	Strongly Disagree	Percentage of Agreements
					5 Scores	4 Scores	3 Scores	2 Scores	1 Scores	
					(A)	(B)	(C)	(D)	(E)	(F = A + B)
S1	3	5	4.72	0.503	74.2	23.4	2.4	0	0	97.6
S2	3	5	4.68	0.486	68.5	30.6	0.8	0	0	99.1
S3	3	5	4.72	0.487	73.4	25.0	1.6	0	0	98.4
S4	3	5	4.77	0.444	77.4	21.8	0.8	0	0	99.2
S5	3	5	4.80	0.423	80.6	18.5	0.8	0	0	99.1
S6	4	5	4.77	0.425	76.6	23.4	0	0	0	100
S7	3	5	4.73	0.483	74.2	24.2	1.6	0	0	98.4
S8	3	5	4.81	0.417	81.5	17.7	0.8	0	0	99.2
S9	3	5	4.71	0.522	74.2	22.6	3.2	0	0	96.8
S10	3	5	4.70	0.525	73.4	23.4	3.2	0	0	96.8
S11	3	5	4.71	0.506	73.4	24.2	2.4	0	0	97.6
S12	3	5	4.77	0.462	78.2	20.2	1.6	0	0	98.4
S13	3	5	4.73	0.479	75.0	23.4	1.6	0	0	98.4
S14	3	5	4.80	0.423	80.6	18.5	0.8	0	0	99.1
S15	4	5	4.80	0.403	79.8	20.2	0	0	0	100
S16	4	5	4.81	0.397	80.6	19.4	0	0	0	100
S17	4	5	4.77	0.425	76.6	23.4	0	0	0	100
S18	4	5	4.83	0.377	83.1	16.9	0	0	0	100
S19	4	5	4.84	0.369	83.9	16.1	0	0	0	100
S20	3	5	4.79	0.428	79.8	19.4	0.8	0	0	99.2

**Table 15:** The measured values of the KMO test and the significance values of Bartlett’s test of the collected questionnaire data of the two scales as listed in Table 13 and Table 14.

Scale	Name of Measure or Test		Value
System usability	KMO measure of sampling adequacy		0.892
	Bartlett test of sphericity	Approx. Chi-Square	818.326
		Degree of freedom	66
		Significance	0.000
User experience	KMO measure of sampling adequacy		0.912
	Bartlett test of sphericity	Approx. Chi-Square	1884.97
		Degree of freedom	190
		Significance	0.000

**Table 16:** Rotated component matrix of the first scale, “system usability”.

	Question Dimension (Scale)	
No.	1	2
T10	**0.813**	0.282
T11	**0.764**	0.285
T12	**0.757**	0.275
T7	**0.741**	0.136
T9	**0.741**	0.398
T8	**0.658**	0.385
T3	0.257	**0.810**
T5	0.258	**0.800**
T4	0.132	**0.732**
T1	0.366	**0.666**
T2	0.330	**0.633**
T6	0.418	**0.577**

**Table 17:** Rotated component matrix of the second scale, “user experience”.

	Question Dimension (Scale)		
No.	1	2	3
S6	**0.723**	0.189	0.276
S19	**0.714**	0.420	0.092
S7	**0.713**	0.289	0.332
S2	**0.681**	0.287	0.379
S20	**0.669**	0.470	0.174
S4	**0.638**	0.317	0.276
S5	**0.629**	0.363	0.185
S3	**0.586**	0.099	0.488
S14	0.198	**0.765**	0.296
S16	0.338	**0.744**	0.266
S15	0.333	**0.739**	0.165
S17	0.534	**0.609**	0.205
S18	0.470	**0.584**	0.359
S13	0.304	**0.582**	0.481
S12	0.355	**0.577**	0.414
S10	0.105	0.280	**0.829**
S9	0.390	0.143	**0.774**
S8	0.226	0.489	**0.622**
S1	0.276	0.340	**0.599**
S11	0.457	0.247	**0.578**

**Table 18:** Collection of questions of the five latent dimensions of the two scales.

Indicator	Question Dimension	Group of Related Questions
System Usability	Perceived Ease of Use (Group FT1)	FT1 = (T10, T11, T12, T7, T9, T8)
	Perceived Usefulness (Group FT2)	FT2 = (T3, T5, T4, T1, T2, T6)
User Experience	Sensation and Emotion (Group FS1)	FS1 = (S6, S19, S7, S2, S20, S4, S5, S3)
	Action and Connection (Group FS2)	FS2 = (S14, S16, S15, S17, S18, S13, S12)
	Cognition and Technology Awareness (Group FS3)	FS3 = (S10, S9, S8, S1, S11)

**Table 19:** The questions of the two scales and the five latent dimensions, as well as corresponding Cronbach’s α coefficients.

Indicator	Question Dimension (Q. D.)	Cronbach’s α Coeff. of Q. D.	Cronbach’s α Coeffi. of Indicator
System Usability	Perceived Ease of Use (Group FT1)	0.889	0.914
	Perceived Usefulness (Group FT2)	0.856	
User Experience	Sensation and Emotion (Group FS1)	0.911	0.956
	Action and Connection (Group FS2)	0.915	
	Cognition and Technology Awareness (Group FS3)	0.867	

**Table 20:** Fitness indexes of the structural models of the two indicators of “system usability” and “user experience” generated through CFA.

Scale	df	χ^2^	χ^2^/df	agfi	cfi	RMSEA	RMSEA (90% CI)	
							LO	HI
System Usability	50	68.814	1.376	0.870	0.976	0.055	0.013	0.085
User Experience	70	183.140	1.308	0.824	0.976	0.050	0.027	0.069

Meanings of symbols—df: degree of freedom; agfi: average gfi; cfi: comparative fit index; RMSEA: root mean square error of approximation; CI: confidence interval; LO: low; HI: high.

**Table 21:** The construct validity values of the latent dimension of the two scales “system usability” and “user experience” generated through CFA.

Indicator	Question Dimension	Group of Related Questions	Construct Validity Value
System Usability	Perceived Ease of Use (Group FT1)	FT1 = (T10, T11, T12, T7, T9, T8)	0.848
	Perceived Usefulness (Group FT2)	FT2 = (T3, T5, T4, T1, T2, T6)	0.849
User Experience	Sensation and Emotion (Group FS1)	FS1 = (S6, S19, S7, S2, S20, S4, S5, S3)	0.891
	Action and Connection (Group FS2)	FS2 = (S14, S16, S15, S17, S18, S13, S12)	0.885
	Cognition and Technology Awareness (Group FS3)	FS3 = (S10, S9, S8, S1, S11)	0.836

**Table 22:** An analysis of responses to questions on the latent dimension of “perceived ease of use”.

No.	Question	Min	Max	Mean	S.D.	Strongly Agree	Agree	No Opinion	Disagree	Strongly Disagree	Percentage of Agreements
						5 Scores	4 Scores	3 Scores	2 Scores	1 Scores	
						(A)	(B)	(C)	(D)	(E)	(F = A+B)
**T10**	Operating the interactive system did not require much mental effort.	4	5	4.71	0.456	71.0	29.0	0	0	0	100
**T11**	I think the operation of the interactive system is very intuitive.	4	5	4.66	0.475	66.1	33.9	0	0	0	100
**T12**	The interaction while operating the interactive system was very smooth.	4	5	4.69	0.463	69.4	30.6	0	0	0	100
**T7**	Learning to use the interactive system was easy for me.	3	5	4.58	0.527	59.7	38.7	1.6	0	0	98.4
**T9**	I think the interface of the interactive system is easy to understand.	3	5	4.71	0.473	71.8	27.4	0.8	0	0	99.2
**T8**	I found that the interactive system made leisure exercise simpler for me.	3	5	4.70	0.494	71.8	26.6	1.6	0	0	98.4

**Table 23:** An analysis of responses to questions on the latent dimension of “perceived usefulness”.

No.	Question	Min	Max	Mean	S.D.	Strongly Agree	Agree	No Opinion	Disagree	Strongly Disagree	Percentage of Agreements
						5 Scores	4 Scores	3 Scores	2 Scores	1 Scores	
						(A)	(B)	(C)	(D)	(E)	(F = A+B)
**T3**	During the experience, it enhanced my hand movements.	3	5	4.77	0.457	79.0	19.4	1.6	0	0	98.4
**T5**	During the experience, it improved the coordination between my eyes, hands, and feet.	3	5	4.80	0.423	80.6	18.5	0.8	0	0	99.1
**T4**	During the experience, it enhanced my leg movements.	3	5	4.73	0.496	75.8	21.8	2.4	0	0	97.6
**T1**	Using this interactive system helps improve my physical health.	3	5	4.75	0.453	75.8	23.4	0.8	0	0	99.2
**T2**	During the experience, it made my leisure exercise easier.	4	5	4.70	0.459	70.2	29.8	0	0	0	100
**T6**	I am willing to continue using the interactive system.	3	5	4.76	0.449	76.6	22.6	0.8	0	0	99.2

**Table 24:** An analysis of responses to questions on the latent dimension of “sensation and emotion”.

No.	Question	Min	Max	Mean	S.D.	Strongly Agree	Agree	No Opinion	Disagree	Strongly Disagree	Percentage of Agreements
						5 Scores	4 Scores	3 Scores	2 Scores	1 Scores	
						(A)	(B)	(C)	(D)	(E)	(F = A+B)
**S6**	During the experience, the interaction energized me.	4	5	4.77	0.425	76.6	23.4	0	0	0	100
**S19**	It showed me how body movements can create leisure experiences with the interactive system.	4	5	4.84	0.369	83.9	16.1	0	0	0	100
**S7**	During the experience, the audiovisual feedback attracted me to continue using it.	3	5	4.73	0.483	74.2	24.2	1.6	0	0	98.4
**S2**	During the experience, it made me feel a sense of friendliness.	3	5	4.68	0.486	68.5	30.6	0.8	0	0	99.1
**S20**	The design of the interactive system made me more willing to engage with technology.	3	5	4.79	0.428	79.8	19.4	0.8	0	0	99.2
**S4**	During the experience, the music gave me a pleasant feeling.	3	5	4.77	0.444	77.4	21.8	0.8	0	0	99.2
**S5**	During the experience, it enhanced my interest in leisure and entertainment.	3	5	4.80	0.423	80.6	18.5	0.8	0	0	99.1
**S3**	During the experience, it brought me a great visual experience.	3	5	4.72	0.487	73.4	25.0	1.6	0	0	98.4

**Table 25:** An analysis of responses to questions on the latent dimension of “action and connection”.

No.	Question	Min	Max	Mean	S.D.	Strongly Agree	Agree	No Opinion	Disagree	Strongly Disagree	Percentage of Agreements
						5 Scores	4 Scores	3 Scores	2 Scores	1 Scores	
						(A)	(B)	(C)	(D)	(E)	(F = A+B)
**S14**	When my friends ask, I will recommend this interactive system.	3	5	4.80	0.423	80.6	18.5	0.8	0	0	99.1
**S16**	If given the chance, I would experience this interactive system again	4	5	4.81	0.397	80.6	19.4	0	0	0	100
**S15**	I would share this experience with my friends.	4	5	4.80	0.403	79.8	20.2	0	0	0	100
**S17**	The interactive system has motivated me to engage in leisure activities.	4	5	4.77	0.425	76.6	23.4	0	0	0	100
**S18**	The interactive system strengthened the connection between leisure activities and physical health.	4	5	4.83	0.377	83.1	16.9	0	0	0	100
**S13**	I would recommend this interactive system to my friends.	3	5	4.73	0.479	75.0	23.4	1.6	0	0	98.4
**S12**	After the experience, I understood the importance of leisure and entertainment.	3	5	4.77	0.462	78.2	20.2	1.6	0	0	98.4

**Table 26:** An analysis of responses to questions on the latent dimension “cognition and technology awareness”.

No.	Question	Min	Max	Mean	S.D.	Strongly Agree	Agree	No Opinion	Disagree	Strongly Disagree	Percentage of Agreements
						5 Scores	4 Scores	3 Scores	2 Scores	1 Scores	
						(A)	(B)	(C)	(D)	(E)	(F = A+B)
**S10**	After the experience, I gained a better understanding of the uses of technology.	3	5	4.70	0.525	73.4	23.4	3.2	0	0	96.8
**S9**	After the experience, I became more willing to try technology.	3	5	4.71	0.522	74.2	22.6	3.2	0	0	96.8
**S8**	During the experience, I felt the entertainment aspect of technology.	3	5	4.81	0.417	81.5	17.7	0.8	0	0	99.2
**S1**	During the experience, the overall interaction was very engaging for me.	3	5	4.72	0.503	74.2	23.4	2.4	0	0	97.6
**S11**	After the experience, it changed my perception of interactive systems.	3	5	4.71	0.506	73.4	24.2	2.4	0	0	97.6

**Table 27:** The record of the user interviews.

Aspect	Question	Record of Interview Comments
**System Interface Operation**	What is your opinion on using body movements for interaction?	(1) The interactive method is interesting and novel, providing a different experience. (P1, P2, P5, P8, P9) (2) It helps the elderly to improve reaction time, physical functions, and brain coordination. (P3, P4, P6) (3) The activities have physical exercise benefits and promote emotional connections. (P7) (4) The system is worth trying and enjoyable. (P10)
	What is your opinion on the operational interface of this work?	(1) The guidance and screen presentation are very clear. (P1, P2, P3, P4, P7, P9) (2) The instructions are clear, easy to understand, and follow. (P5, P6, P10) (3) The simple interface made the interaction smooth. (P8)
	Was the process of learning and operating this work smooth for you?	(1) Most respondents felt the operation process was smooth. (P1, P2, P4, P5, P6, P8, P9) (2) Respondents were able to quickly adapt and operate proficiently. (P1, P5, P6, P7, P9) (3) Initially, there was some difficulty, but it became smoother with practice. (P3, P10) (4) The overall experience is satisfactory and pleasing. (P7)
**Experience Feelings**	What is your opinion on the exhibition setup or digital content design?	(1) The design was highly satisfactory, interesting, and suitable for the elderly. (P1, P2, P5, P6, P8, P9, P10) (2) The elderly benefit from the design, with a positive impact on body movements. (P3, P4) (3) The staff were friendly, and the setup and game content enhanced the entertainment value. (P7)
	Did you feel happy during the interactive experience? Why?	(1) Respondents generally felt very happy during the interaction process. (P1, P2, P4, P5, P8, P9, P10) (2) The novel ball and tennis games brought joy. (P3, P1) (3) Learning new skills and achieving high scores increased satisfaction. (P6, P7)
	Do you have any other thoughts or feelings after the interactive experience?	(1) The experience left respondents feeling satisfied and happy. (P1, P5, P6, P10) (2) Respondents hope for more such activities and games in the future. (P3, P4) (3) They expect the activities to be held more frequently and be beneficial to physical health. (P7, P9)
**Views on AI Integration in Elderly Exercise and Leisure**	What is your opinion on applying AI technology to leisure and entertainment?	(1) AI technology makes leisure activities more interesting and innovative. (P1, P3, P4, P8, P9, P10) (2) The introduction of technology increases interactivity and diversity in the experience. (P2, P5) (3) It promotes physical movement and enhances the sense of participation in entertainment. (P6) (4) The elderly are open to new technology and look forward to more opportunities to experience it. (P7)
	Has AI technology increased your willingness to engage in leisure and entertainment activities?	(1) Most respondents said that the technology increased their willingness to participate. (P1, P2, P3, P4, P5, P6, P7, P8, P9) (2) The introduction of technology effectively stimulated the elderly’s interest and participation motivation. (P3, P7, P1) (3) They hope for more similar activities in the future to enhance the participation experience. (P10)

## Data Availability

The datasets used and/or analyzed during the current study are available from the corresponding author on reasonable request.
